# Eco-Interactions of Engineered Nanomaterials in the Marine Environment: Towards an Eco-Design Framework

**DOI:** 10.3390/nano11081903

**Published:** 2021-07-24

**Authors:** Ilaria Corsi, Arianna Bellingeri, Maria Concetta Eliso, Giacomo Grassi, Giulia Liberatori, Carola Murano, Lucrezia Sturba, Maria Luisa Vannuccini, Elisa Bergami

**Affiliations:** 1 Department of Physical, Earth and Environmental Sciences, University of Siena, Via P. A. Mattioli 4, 53100 Siena, Italy; arianna.bellingeri@student.unisi.it (A.B.); maria.eliso@student.unisi.it (M.C.E.); giacomo.grassi@student.unisi.it (G.G.); giulia.liberatori@student.unisi.it (G.L.); carola.murano@student.unisi.it (C.M.); lucrezia.sturba@student.unisi.it (L.S.); marialuisa.vannuccini@unisi.it (M.L.V.); elisa.bergami@unisi.it (E.B.); 2 Department of Biology and Evolution of Marine Organisms, Stazione Zoologica Anton Dohrn, Villa Comunale, 80121 Naples, Italy; 3 Agro Paris Tech, Université Paris-Saclay, INRAE, UMR ECOSYS, 78026 Versailles, France; 4 British Antarctic Survey, Natural Environment Research Council, High Cross, Madingley Road, Cambridge CB3 0ET, UK

**Keywords:** marine pollution, engineered nanomaterials, nanoecotoxicology, bio-nano interactions, behavior, titanium dioxide, silver nanoparticles, polystyrene nanoparticles, eco-safety, eco-design

## Abstract

Marine nano-ecotoxicology has emerged with the purpose to assess the environmental risks associated with engineered nanomaterials (ENMs) among contaminants of emerging concerns entering the marine environment. ENMs’ massive production and integration in everyday life applications, associated with their peculiar physical chemical features, including high biological reactivity, have imposed a pressing need to shed light on risk for humans and the environment. Environmental safety assessment, known as ecosafety, has thus become mandatory with the perspective to develop a more holistic exposure scenario and understand biological effects. Here, we review the current knowledge on behavior and impact of ENMs which end up in the marine environment. A focus on titanium dioxide (n-TiO_2_) and silver nanoparticles (AgNPs), among metal-based ENMs massively used in commercial products, and polymeric NPs as polystyrene (PS), largely adopted as proxy for nanoplastics, is made. ENMs eco-interactions with chemical molecules including (bio)natural ones and anthropogenic pollutants, forming eco- and bio-coronas and link with their uptake and toxicity in marine organisms are discussed. An ecologically based design strategy (eco-design) is proposed to support the development of new ENMs, including those for environmental applications (e.g., nanoremediation), by balancing their effectiveness with no associated risk for marine organisms and humans.

## 1. Introduction

Along with social and economic development, population growth and the increasing interests in exploitation and utilization of marine resources, a progressive increase in marine pollution has been encountered. Marine communities in coastal areas and in the deep sea are continuously threatened by various classes of chemical pollutants with associated environmental deterioration and reduction in ecosystem services [1]. Since the sustainable development and ecological protection of the oceans mean that of humans, pollution monitoring and risk assessment are mandatory [2]. With the aim to achieve a Good Environmental Status in European marine waters [3] and meet the target posed by UN Sustainable Development Goal for the oceans (SDG 14, Life below water), actions aimed to prevent, remedy, monitor and report on ocean pollution are urgently needed (EC Action Plan: ‘Towards Zero Pollution for Air, Water and Soil’) [4]. Although several anthropogenic pollutants, which constitute a menace for the oceans worldwide and in turn for human health (One Health) [5], are now managed through specific regulatory and mitigation actions, contaminants of emerging concerns (CECs) are far less investigated despite the hazard posed by them to humans and the environment.

Among CECs, engineered nanomaterials (ENMs, size range 1–100 nm, [6]) are globally recognized as a major threat for the marine environment [7]. Their production, usage and disposal are exponentially increasing worldwide. Yet, ENMs are unmonitored and unregulated, thus their amounts in the environment can approach acute toxicity thresholds with adverse effects on humans and marine wildlife [8,9]. During their synthesis, peculiar desired properties can be tightly controlled, thus providing them unique chemical and physical properties, which are drivers of their behavior in environmental compartments [10]. ENMs are mostly incorporated into liquids or solids to form nano-enabled products (NEPs) with numbers exceeding 300,000 items and 24,000,000 patents in 2020 (http//www.nano.nature.com, accessed on 17 June 2021). Such massive production and integration in everyday life applications, associated with unique physical and chemical features including high biological reactivity, have imposed a pressing need to shed light on environmental and human health risk assessment. From diverse sources they are released in air, soils and aquatic compartments from which they end up into the marine environment.

Material flow and fate modelling originating from a life-cycle concept analysis which include all environmental compartments, key transformation processes (e.g., aggregation, sedimentation, dissolution) and direct and indirect environmental emissions, indicate that ENMs could escape to the environment during manufacture (e.g., disposal in manufacturing plant wastewaters; fugitive emissions), use (attrition and weathering) and disposal and finally reach environmental sinks as marine sediments [9].

Based on a dynamic material-flow analysis model, calculated input of masses taking into account process-based descriptions of ENMs fate and behavior in natural systems and accurate mass input parameters identified nanoscale titanium dioxide (n-TiO_2_) as reaching far higher concentrations than other ENMs, with the worst-case scenario in marine sediments of about 44,000 tons compared to 30 tons for silver nanoparticles (AgNPs). Despite such huge differences in environmental concentrations, n-TiO_2_ and AgNPs show a similar pattern with sludge, waste and wastewater effluents considered flow-through compartments with no accumulation while sediment and soil act as the accumulating final sinks [11].

Emissions of other ENMs, such as organic polymeric NPs, from commercial applications and consumer products are expected to reach far lower quantities in the environment than n-TiO_2_ and AgNPs [12]. However, input from secondary sources should also be considered, as in the case of nano-sized polymers, including polystyrene (PS) NPs, which can be generated by 3D printing [13] and fragmentation of large plastic debris [14,15]. As the global production of plastics reached 368 million tonnes in 2019 [16], dumping of plastic waste and discharge of products containing micro- and nanoplastics in the environment is increasing and projected to reach up to 155–265 million metric tonnes per year by 2060 [17]. Although knowledge gaps remain that must be filled in order to model their environmental fate, PS NPs have recently been traced in wastewaters associated with biosolids [18] and in estuarine and marine surface waters [19,20], raising concerns over their potential distribution through the water column and accumulation in the seabed.

Therefore, ENMs environmental safety assessment known as “eco-safety” has become a requirement to gather a more holistic view of exposure scenarios and consequent biological effects [8].

Here, we review the current knowledge on ENMs which end up in the marine environment, environmental transformation occurring in seawater and their effects documented on marine biota at different trophic levels. A focus on those ENMs considered to reach high exposure concentrations for marine organisms, as n-TiO_2_ and AgNPs, among metal-based ENMs massively used in commercial and consumer products, and polymeric NPs as PS, largely adopted as proxy for nanoplastics, has been made. AgNPs embrace more peculiar nano-based properties, regardless of chemical core composition, such as surface charge, dissolution and adsorption, all dictating their eco–bio interactions in marine waters, uptake and toxicity for marine organisms from different trophic levels, up to ecological functioning. ENMs behavior in marine waters as well as interactions with chemical molecules including (bio)natural molecules and those of anthropogenic origin (i.e., marine pollutants), forming eco- and bio-coronas which drive their uptake and toxicity in marine organisms are described. A wide range of negative effects caused by ENMs exposure are also reported across a range of marine species and at different levels of complexity, from (sub-)cellular to single individual up to population level. An ecologically based design strategy (eco-design) is finally proposed to support the development of new eco-safe ENMs by balancing their effectiveness with no associated risk for marine organisms and humans. Therefore, not only implications but also scenarios of environmentally safe future applications of ENMs are critically illustrated as for instance innovative eco-safe nano-based solutions to monitor and mitigate the presence and the hazard posed by legacy pollutants into the marine environment.

## 2. Behavior and Exposure Assessment of ENMs in Marine Waters

**Figure 1:**
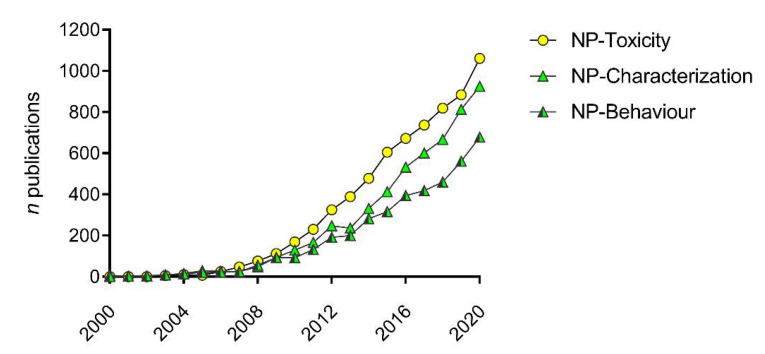
The importance of ENM/NP behavior (green) in nano-ecotoxicity studies (yellow) in the last 20 years (2010–2020). Data obtained from Scopus, search queries: ((TITLE-ABS-KEY(nanoparticles OR nanomaterials AND behavior) AND DOCTYPE(ar) AND PUBYEAR > 1999 AND PUBYEAR < 2021) AND (LIMIT-TO (SUBJAREA,”ENVI”) OR LIMIT-TO (SUBJAREA,”AGRI”)))((TITLE-ABS-KEY(nanoparticles OR nanomaterials AND characterization) AND DOCTYPE(ar) AND PUBYEAR > 1999 AND PUBYEAR < 2021) AND (LIMIT-TO (SUBJAREA,”ENVI”) OR LIMIT-TO (SUBJAREA,”AGRI”)))((TITLE-ABS-KEY(nanoparticles OR nanomaterials AND toxicity) AND DOCTYPE(ar) AND PUBYEAR > 1999 AND PUBYEAR < 2021) AND (LIMIT-TO (SUBJAREA,”ENVI”) OR LIMIT-TO (SUBJAREA,”AGRI”))).

Extensive scientific research has been produced since 2000 to gather knowledge on the behavior and (eco)toxicity of ENMs. By searching the words “nanoparticles/nanomaterials” and “toxicity” on the Scopus database, over 6500 research articles in the field of environmental sciences are found, with the vast majority published in the last decade (95.8% from 2010 to 2020) (Figure 1). If the same search is carried out using the words “nanoparticles/nanomaterials” and “characterization/behavior”, a similar increasing trend is found, underlining the intrinsic connection between the behavior and effects of ENMs and the importance of such knowledge in ecotoxicological studies and environmental risk assessment (Figure 1).

ENMs are extremely diverse in terms of core composition, size, shape, and surface coating, which also affects their reactivity towards living organisms; their behavior in environmental compartments, and in particular, in the aquatic compartments, is first dictated by specific combinations of those properties. For instance, the surface charge of polymeric NPs has been associated with distinct biological responses in marine organisms [21,22,23], while, as far as metal-based ENMs, crystal morphology (i.e., n-TiO_2_) and dissolution (i.e., AgNPs) have been correlated to their ecotoxicity [24,25,26].

**Figure 2:**
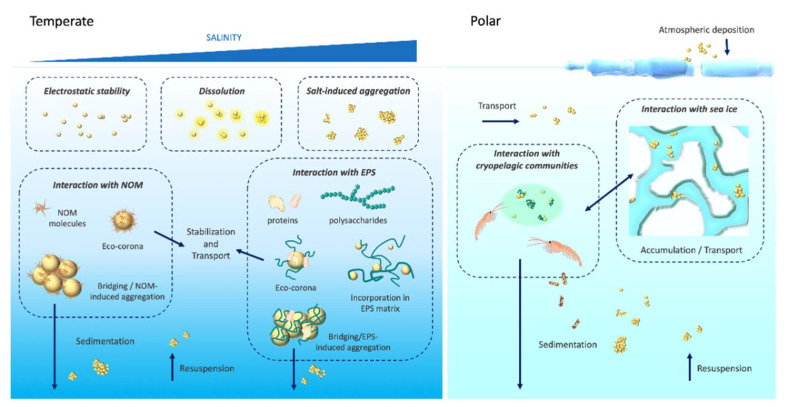
Schematic showing the processes regulating ENMs behavior, transport and fate in the marine environments from temperate (**left**) to polar (**right**) regions. Left: ENMs stability, dissolution, interaction with NOM and EPS and eco-corona formation are shown at increasing salinity. Right: ENM accumulation and transport in sea ice and interaction with cryopelagic communities are shown. Not to scale.

Environmental transformations such as those occurring in marine waters are the result of manifold interactions with abiotic (e.g., water chemistry, ionic species, colloids) and biotic (e.g., exudates from microbiome and plankton) components [27], reflecting the high variability of complex natural settings and how they interact with ENMs (Figure 2).

Nano-ecotoxicology has focused on single and/or multiple factors on a case-by-case studies in order to disentangle the risk posed by ENMs based on their selective properties and of those of the receiving media. First investigations aimed at defining exposure conditions to address ENM behavior in artificial media, such as NaCl solutions or reconstituted artificial seawater, following standardized protocols for ecotoxicity testing.

Changes in ENM intrinsic properties in response to selected abiotic variables, such as pH, temperature, single ions and commercially available humic substances, have been investigated providing clues on their transformation once dispersed in complex natural aquatic media [28,29].

Overall, findings underlined the importance of a detailed physical and chemical characterization of bare ENMs in exposure media. This aspect has been recognized as fundamental to unravel nano-bio-interactions (i.e., transformations) and link exposure to the observed biological effects. For this purpose, environmental media such as natural waters (e.g., collected from the field and filtered), have recently been adopted to examine more realistic environmental conditions and mimic real exposure scenarios [30]. In our recent paper, we have reviewed the importance of such eco-interactions for ENMs entering the aquatic systems as drivers of fate and ecotoxicity for marine organisms [31].

### 2.1. The Role of ENMs Surface Charges

The behavior of ENMs entering the marine environment could change as a function of their intrinsic chemical signature but also of physical and chemical properties of the receiving water bodies (i.e., temperature, osmolarity, pH, etc.).

In temperate marine waters (i.e., surface water temperature ranging between 16 and 25 °C, salinity of 38–41‰), ionic strength has been shown as the major driver of agglomeration of NPs bearing a surface charge. Regardless of their chemical core composition and size, the presence of negative charges on the NP surface dictates the formation of micron-sized agglomerates soon after suspension in seawater with destabilization of the particle surface (i.e., n-TiO_2_ Aeroxide© P25, 25 nm; carboxylated PS NPs (PS-COOH), 40 nm) [21,22,32,33]. On the contrary, the presence of positive charges prevents such strong and quick agglomeration (i.e., cationic amino-modified groups on PS NPs, PS-NH_2_) and allows them to be initially present as nanoscale entities thus more easily taken up by exposed cells/organisms [21,22,23,32,34]. In turn, the formation of micrometric agglomerates influences NP buoyancy, with large agglomerates settling in exposure vessels and becoming presumably more bioavailable for benthic species in natural exposure scenarios [32,33,34,35]. The theoretical description behind the behavior of charged NPs is the Derjaguin–Landau–Verwey–Overbeek theory and following modifications [36,37,38], which describe the interactions of the electrostatic repulsion forces governing NP stability. However, among the investigated physical and chemical parameters of the receiving waters, how temperature might affect NP behavior in natural scenarios has been little studied so far.

In polar marine environments, low sea temperature (<0 °C) has been indicated among the critical factors that may significantly influence the behavior and fate of ENMs (Figure 2), together with seasonal changes in UV radiation, natural organic matter (NOM) and sea ice formation. Although studies in polar areas are still limited, probably being considered far from the major emission sources of ENMs, long-range atmospheric and oceanic transport could deliver them to high latitudes as shown for larger polymeric particles [39,40,41] and organic nanogels in the Arctic [42]. Local sources due to the presence of scientific research stations and remote communities may also contribute to local emissions of incidental ENMs, through combustion processes and wastewaters and/or sewages (i.e., personal care products, sunscreen, etc.) [43]. With the increasing exploitation of polar environments, contamination from ENMs is likely to become a major environmental issue both in the Arctic and in the Southern Ocean (e.g., Antarctica) [44]. Our recent findings on PS NPs in seawater suspensions under Antarctic-like conditions (0 °C, 34‰, pH 7.98) [45] showed a clear difference in the NP dispersion with respect to data obtained for temperate regions (>18 °C, 38‰, pH 8.30) [21,22]. The initial low agglomeration of PS-COOH NPs in Antarctic seawater was associated with the uptake and toxicity in the Antarctic sea urchin (*Sterechinus neumayeri*) coelomocytes upon in vitro exposure (6–24 h) [45]. Since NP agglomeration is mainly related to NP Brownian motion and directly associated to the temperature of the system [46], high temperatures correspond to high reactivity and collision frequency of NPs, with consequent high agglomeration [37]. Under polar environmental conditions, sea temperatures usually range between −1.8 and 4 °C over one year, with an expected low agglomeration rate. A first attempt in mimicking NP interactions with sea ice through a freeze–thaw cycle (from −20 to 0 °C) also revealed irreversible agglomeration and instability of PS NPs, determined by Dynamic Light Scattering (DLS) analysis [45]. Sea ice is a well-known temporal sink and vector of many contaminants, including anthropogenic particles (i.e., micron-size plastics), which can reach concentrations that are several orders of magnitude higher than in the surrounding waters [39]. The interaction with extracellular polymeric substances (EPS) released from sea ice algae could also favor this process [47]. Similarly, once ENMs reach polar regions, they can be trapped in brine channels during the sea ice formation (Figure 2), with unknown repercussions on their physical and chemical state, redistribution, and impact to the biota, once sea ice melts. Polar cryopelagic communities, which also include sea ice algae and euphausiid larvae, strictly depend on sea ice and may be particularly exposed to ENMs through adsorption and/or ingestion. The Antarctic krill (*Euphausia superba*), a keystone species of marine ecosystems in the Southern Ocean, has been found to feed on plastic microspheres and fragment them to sub-micron and likely nanoscale objects through digestion [48]. We have further shown that PS NPs could be ingested by krill *E. superba* juveniles upon short-term exposure and incorporated in fecal pellets [49]. From an ecological perspective, the incorporation of anthropogenic NPs in the feces of zooplankton may affect the sinking of biogenic material to the ocean sea floor, its remineralization, and alter the natural biogeochemical cycles [50]. Therefore, more studies are necessary to characterize the behavior of ENMs in polar environments and the role of surface charge to obtain a comprehensive view of their fate and impacts in such unique marine environmental conditions. ENMs and their surface charge appear as highly dynamic structures in seawater which indeed require a closer look due the huge variability of physical chemical parameters encountered in marine waters from estuarine, brackish and marine coastal environments to the deep sea and polar regions [51,52].

### 2.2. The Interplay with Dissolved/Particulate NOM and Existing Chemicals in Seawater

**Figure 3:**
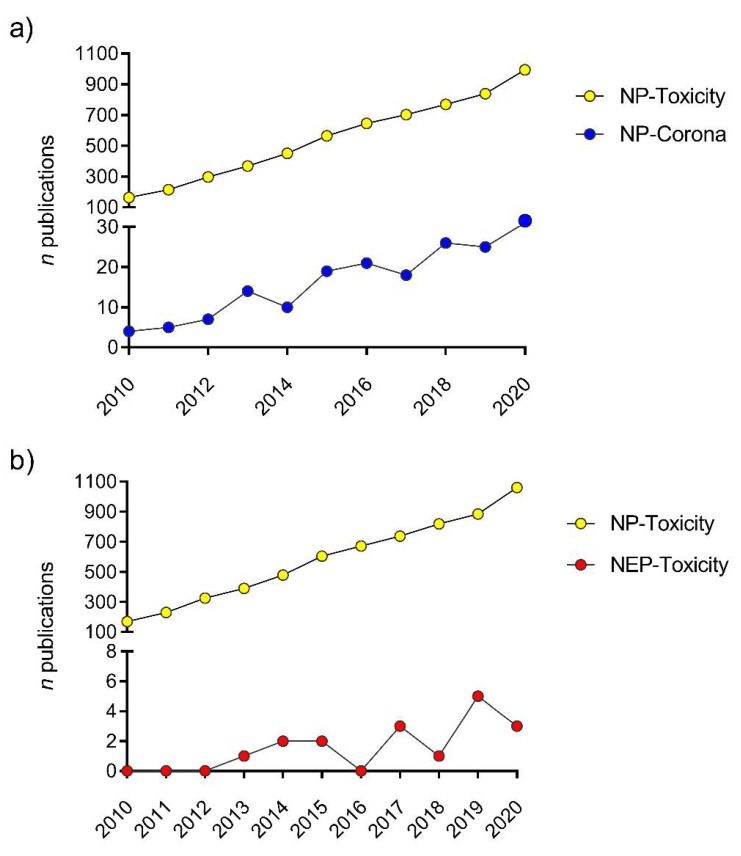
(**a**) The increasing relevance of NP eco- and bio-coronas (blue) in nano-ecotoxicity studies (yellow) published in the period 2010–2020. Data obtained from Scopus, search queries: ((TITLE-ABS-KEY(nanoparticles AND toxicity) AND DOCTYPE(ar) AND PUBYEAR > 1999 AND PUBYEAR < 2021 AND (LIMIT-TO (SUBJAREA,”ENVI”) OR LIMIT-TO (SUBJAREA,”AGRI”))); ((TITLE-ABS-KEY(nanoparticles AND corona) AND DOCTYPE(ar) AND PUBYEAR > 1999 AND PUBYEAR < 2021 AND (LIMIT-TO (SUBJAREA,”ENVI”) OR LIMIT-TO (SUBJAREA,”AGRI”))). (**b**) Ecotoxicity studies conducted using engineered nanoparticles/nanomaterials (yellow) and nano-enabled products (red) in the period 2010–2020. Data obtained from Scopus, search queries: ((TITLE-ABS-KEY(nanoparticles OR nanomaterials AND toxicity) AND DOCTYPE(ar) AND PUBYEAR > 1999 AND PUBYEAR < 2021) AND (LIMIT-TO (SUBJAREA,”ENVI”) OR LIMIT-TO (SUBJAREA,”AGRI”))); ((TITLE-ABS-KEY (nano-enabled AND toxicity) AND DOCTYPE (ar) AND PUBYEAR > 1999 AND PUBYEAR < 2021 AND (LIMIT-TO (SUBJAREA,”ENVI”) OR LIMIT-TO (SUBJAREA,”AGRI”))).

All aquatic environments are characterized by the presence of dissolved/particulate NOM, whose concentrations and composition can affect ENMs behavior and stability, for example, via the formation of clusters and bridging effects. However, the role of NOM can be prevalent in freshwater and estuarine environments, whose content is notably higher than in marine waters where it is outshone by the impact of the high ionic strength [23,36,51] and the presence of EPS (Figure 2). Due to their peculiar high surface to volume ratio, ENMs easily interact with a wide range of biomolecules which they come across in natural aqueous media [53]. Dissolved or dispersed, they can range from allochthonous ones, deriving from the degradation of terrestrial biomass, to the ones produced by planktonic organisms. As such, their interactions with ENMs can substantially differ and originate a wide variety of different new nanosized objects fundamentally reshaped and bearing newly acquired properties with which they are presented to biological systems ([31] and reference within). For instance, such interplay could involve proteins, carbohydrates, and metabolites [54] including nucleic acids whenever they encounter biological entities [55]. Originally, the adsorption of a protein layer at the NP surface, representing the outmost contact point between the nanosized objects and biological membranes, represented the basis for the conceptualization of the *protein corona* paradigm [56]. As such, this newly acquired protein coating confers to the NP a new biological identity, distinct from the inherent material and provides an important pattern for its interactions with biological systems [57]. Different facets of this topic have been identified and studied over the past two decades. Firstly, this biomolecular signature was exploited in the field of medical biotechnology for the advanced drug delivery targeting specific cell types and avoiding immune clearance, and to tune the desired NP–cell interactions [58,59]. Consequently, the protein corona became an important clue to understand and decipher the novel toxicological challenges posed by undesired contact of nanosized objects with living systems. Nanotoxicological research benefited from incorporating the study of the composition of biomolecular coronas, as adsorbed proteins can contribute to increase or alleviate NP toxicity [60,61] (Figure 3a).

The presence of certain proteins can promote the recognition by specific cellular receptors, hence determining the biological fate of NPs [62]. Uptake by endocytic or phagocytic pathways is often observed and can greatly determine the effects sustained by cells [63]. For instance, the presence of some epitopes or specific protein patterns, such as damage associated molecular patterns (DAMPs) can direct the intracellular fate of NPs. Interestingly, it was observed that the formation of such patterns due to protein unfolding after adsorption on positively charged NPs (PS-NH_2_) led to interactions with scavenger receptors, while the same protein in its native state in the corona of negatively charged ones (PS-COOH) channeled their interactions with cells via albumin receptors [64,65], possibly triggering a different cytotoxic response. Wan and co-authors [66] have demonstrated that the de-glycosylation of corona proteins critically affects NP adsorption and uptake by macrophages and the generation of a pro-inflammatory cellular environment, compared to a fully glycosylated corona. Additionally, when NPs enter biological milieus, their physical chemical status tends to change remarkably. The adsorption of biomolecules was demonstrated to either improve colloidal stability of NPs by increasing the electrostatic barrier to aggregation, or to destabilize well-dispersed NP suspensions due to, for instance, bridging mechanisms or surface charge saturation [67,68,69,70]. Taken together, these aspects associated with the interactions between NPs and biomolecules delineate variables that need to be taken into account when approaching the (eco)toxicity study of nanosized objects, as they interact with living entities in a fundamentally different way than classical contaminants [71,72]. While biotechnology and toxicology have already incorporated different aspects of the biomolecular corona in studies with NPs, such concepts made their way in the field of nano-ecotoxicology only recently by incorporating the systematic characterization of NP coronas [8,73,74].

**Figure 4:**
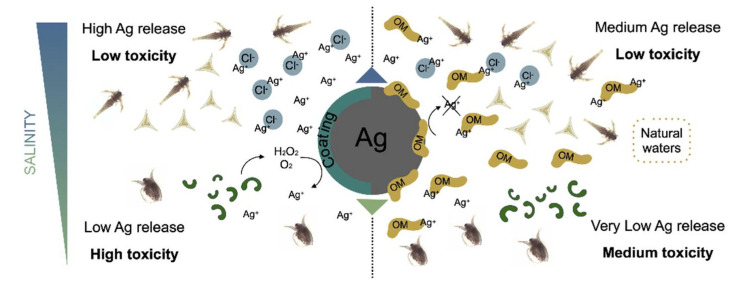
Release of Ag^+^ ions from AgNPs and toxicity on aquatic plankton as influenced by environmental conditions and water chemistry (OM: Organic Matter). Not to scale.

Indeed, in the marine environment, the study of coronas proved important for a thorough understanding of NP fate and behavior. However, in natural seawater, the study of environmental coronas, named eco-corona [75], acquires another dimension due to numerous complicating variables, normally not present in in vitro experimental conditions [76]. Therefore, an extension of the corona paradigm to incorporate the ecotoxicological perspective of environmental nano–bio interactions is needed [77]. This means to deeply focus on the eco-corona concept, as the adsorption of dissolved organic molecules making up the NOM pool of seawater, such as refractory organic matter (i.e., fulvic and humic acids) and EPS (i.e., polysaccharides, proteoglycans and proteins) (Figure 4). The already intricate scenario for the adsorption of such a heterogeneous spectrum of biomolecules on the NPs is complicated by the complexity of the water medium, suggesting that different outcomes are expected and that a thorough examination of the variables involved is necessary.

Firstly, working with ecologically significant marine organisms means dealing with the biology of a non-model species. It follows that this can hamper the study of biomolecular coronas formed in the biological milieus of such species. Nevertheless, when such an approach is enforced, it allows a deeper understanding of the observed effects.

The formation of an eco-corona greatly influences the behavior of NPs in seawater; an increase in colloidal stability has often been observed by adding commercially available NOM fractions to NP dispersions in seawater, in conditions ensuring a high surface coverage [78]. In this complex scenario, dissolution of metal-based ENMs such as AgNPs further complicates the knowledge of exposure conditions and interplay with existing biomolecules. AgNPs are recognized as being one the most widely used type of NPs in consumer products, especially for their efficient antimicrobial properties [79]. The main feature influencing the toxicity of AgNPs is their ability to release Ag^+^ ions [80], known as some of the most toxic metal ions in the aquatic environment [81] (Figure 4).

However, information concerning this issue is often conflicting, with some studies assigning all observed toxic effects to dissolved Ag^+^, while others reporting an additional toxicity probably linked to the nano-size. It is interestingly pointed out by Yang and co-authors [82] how most studies reporting a dissolved Ag^+^-related toxicity are performed on multicellular organisms, while those claiming additional effects usually focus on single cell species. Direct contact of AgNPs with cell membranes often results in the production of exudates with oxidative potential, or even cellular internalization, thereby leading to a possible additive toxicity.

The dissolution of AgNPs firstly depends on intrinsic features of the particles, but also, and to a great extent, on extrinsic ones, such as physical chemical properties of the receiving environment. AgNPs are one of the best examples of how experimental and environmental conditions are able to completely change the behavior and consequent toxicity of ENMs [83,84]. A partial control of ion release can be obtained with surface functionalization. In the literature various types of surface functionalized AgNPs have been described and associated with a highly variable degree of particle dissolution [85,86,87,88].

Surface coating reduces the particle’s contact with oxidizing agents, such as dissolved oxygen and reactive oxygen species (ROS), reducing the chances and the degree of dissolution. It also plays an indirect role, by driving aggregation and surface charge which, in turn, influence the extent of the surface available for dissolution and the interaction with other molecules and living organisms. As an example, reduced dissolution and toxicity have been documented with surface coatings made by sulfur or sulfur-containing molecules [89] such as cysteine [90].

The physical chemical characteristics of the receiving aquatic environment is also crucial in determining the dissolution of AgNPs. Using laboratory synthetic media, Lish and co-authors [83] observed that an increase in salinity corresponded to an increase in Ag^+^ ions release. However, this was not followed by an enhanced toxicity. Ag^+^ ions are in fact easily bound by chloride (Cl^−^) species, which are abundant in seawater, thus reducing their bioavailability and uptake by organisms. Cl^−^ is considered a weak Ag ligand, whose influence on AgNPs dissolution, Ag^+^ bioavailability and consequent toxicity, may vary according to Ag/Cl ratio, leading to non-linear biological responses with sometimes more severe effects at lower Ag concentrations [91,92].

The presence of oxidizing agents, such as dissolved oxygen and free Ag ligands, as well as other external factors such as low pH, can trigger the dissolution of AgNPs. Hydrogen peroxide (H_2_O_2_), which can also be produced by organisms together with other oxidizing molecules, was shown to greatly enhance the dissolution of AgNPs [93]. Cysteine, a strong metal ligand, in some cases, was observed to induce the dissolution of AgNPs when free in solution, with various degrees of efficiency [85,93]. On the other hand, NOM rich in sulfur and nitrogen, such as humic and fulvic acids, plays a crucial role in natural waters against AgNPs dissolution [94], which results in a reduced toxicity [95]. As AgNPs dissolution depends on many different factors, and environmental/experimental conditions, the understanding of their behavior in exposure media appears to be the best tool for predicting their environmental impact and associated risk for biota. However, dissolution rate and toxicity do not always come together, as released ions often undergo chemical transformations depending on water chemistry, reducing their bioavailability, as demonstrated by Lish and co-authors [83]. In other circumstances, toxicity seems to be the result of more complex dynamics, being higher than expected based on AgNPs dissolution rate, leading to the hypothesis of a nano-specific toxicity involvement [96,97]. Although a significant number of studies aimed at clarifying the behavior of AgNPs in exposure media linking them to the observed cytotoxic effects, very little research has conducted in natural seawater in order to unravel the potential interplay with biomolecules and how this might affect dissolution and consequent ecotoxicity (Figure 4).

### 2.3. Nano–Eco Interactions Scenarios Leading to Ecotoxicity

Nano–eco interactions, such as the formation of an eco-corona around NP when in contact with natural seawater media, can significantly trigger ENMs toxicity to marine species. Mitigation of toxicity was observed for PS [98,99] and metal-oxide [100] NPs, essentially due to the buffering effect of the eco-corona limiting for instance, NP surface reactivity and/or dissolution, ultimately providing a barrier to the direct contact with biological membranes. Concerning the EPS-formed corona, their influence on NP colloidal behavior has been demonstrated. In our previous work, n-TiO_2_ colloidal stability upon the exposure to EPS produced by the marine alga *Dunaliella tertiolecta* was investigated, underlying a decrease in aggregation and sedimentation rate compared to bare NP; thus prospecting a different fate for n-TiO_2_ following eco-corona formation in seawater [101]. Moreover, the protein pattern of n-TiO_2_ eco-corona differed from the total EPS pool of *D. tertiolecta*, suggesting an adsorption specificity. Similarly, the dynamic formation of an EPS eco-corona was later investigated in depth with the EPS from the marine diatom *Phaeodactylum tricornutum* and negatively charges PS NPs (PS-COOH), revealing a preferential interaction with certain compounds over a wide range of molecular weights. Mainly high molecular weight polysaccharides and low molecular weight proteins were found adsorbed onto PS-COOH NP, out of the highly chemical heterogeneous continuum of *P. tricornutum* EPS. In turn, microscopic examination revealed the structure of the NP-EPS complexes, with a peculiar distribution of carbohydrates and proteins in the aggregates, rethinking the corona concepts in natural waters [102].

Similarly, in the work with Canesi and co-workers [103], a soluble protein from the serum hemolymph of the marine mussel *Mytilus galloprovincialis* was identified in the protein-corona around amino-modified PS NPs (PS-NH_2_) and found as the discriminant element for the observed toxicity. In the sea urchin *Paracentrotus lividus*, the composition of the biomolecular corona acquired in the coelomic fluid by the same PS-NH_2_ NPs was studied [104]. The main component was the Toposome precursor, a calcium-binding transferring-like protein, whose documented functions involve cell adhesion and clotting, embryogenesis and immune response. Selective accumulation in phagocytic coelomocytes and cytotoxic effects due to exposure to PS NPs greatly diminished when the Ca^2+^ was depleted from the coelomic fluid by the addition of a chelating agent as EDTA, thus confirming the facilitating effect of the Toposome in NP–coelomocytes interactions and consequent cellular damages. In parallel, a proteomic approach was applied by also including PS-COOH NPs as a negatively charged counterpart. Similar protein patterns were identified on both PS NP regardless of their different surface charge, thus not only confirming the presence of Toposome adsorbed on PS-COOH NPs, but also suggesting a similar internalization mechanism. Interestingly, other proteins with a similar function were identified, such as flotillin [105]. Moreover, KEGG pathway enrichment analysis of the identified corona proteins revealed their involvement in phagosome formation processes, strengthening the hypothesis of corona favoring the interaction with *P. lividus* phagocytic cells. Through molecular character, such findings indicating how the effects of NPs on the sea urchin immune system are exerted can be an interesting propeller to comprehend the effects rebounding at the organism and community levels, due to the contamination by nanosized PS in marine waters.

Another relevant scenario in NP–surface interaction is with existing marine pollutants, which could affect their bioavailability and increased/reduced toxicity to marine organisms. Proposed by Limbach and co-authors in 2007 [106], the ability of ENMs to act as *Trojan horse* carriers upon co-incubation with selected legacy pollutants and CECs, and consequent differential bioaccumulation and/or toxicity compared to single chemical/ENM exposure, has been demonstrated [107].

Our findings on the interplay between Cd^2+^ and n-TiO_2_ (at 0.1 and 1 mg L^−1^, respectively) showed a significant reduced ability of n-TiO_2_ (pure anatase) to adsorb Cd^2+^ ions in both natural and artificial seawater, contrarily to suspensions in ultrapure water in which high adsorption efficacy of Cd^2+^ into n-TiO_2_ surface was observed. DLS analysis further revealed a significant reduction in negative ζ-potential of n-TiO_2_ in seawater suspensions compared to ultrapure water probably due to the high ion concentration which suppresses the electrostatic repulsion of negative charges; this could act by accelerating homoaggregation (i.e., aggregation of NPs among themselves) and thus reducing its ability to bind Cd^2+^ [33]. Similarly, we [108] reported no adsorption of Cu^2+^ ions on negatively charged PS–COOH NPs upon incubation in an algal medium naturally enriched with dissolved ions compared to ultrapure water. On the other hand, the sorption ability towards chemicals such as persistent organic pollutants (POPs) as well as specific hydrocarbons (i.e., phenanthrene, PhE) onto the NP surface has been demonstrated in other studies, thus stimulating the need to unravel specific surface mechanism rather than chemical core composition behind such observed chemical selectivity [109,110].

An antagonistic role of n-TiO_2_ towards Cd chloride (CdCl_2_) was in fact observed in Cd-induced genotoxicity in the marine mussel *M. galloprovincialis* upon in vivo exposure [111,112] as well as a reduction in Cd bioaccumulation in the gills and whole soft tissues [33]. Moreover, the presence of n-TiO_2_ reduced other Cd-induced specific effects by significantly lowering *abcb1* gene transcription, Glutathione S-transferases activity, whereas additive effects were observed on nitric oxide production [33].

Contradictory findings were observed in combining the exposure to n-TiO_2_ and 2,3,7,8-tetrachlorodibenzo-*p*-dioxin (TCDD) (0.1 mg L^−1^ and 0.25 µg L^−1^, respectively) both in vitro and in vivo [113]. In mussels in vivo exposed to the mixture, a decrease in the histological alterations induced by TCDD alone in the digestive gland was observed while an increase in TCDD levels were found [112].

The absence of a *Trojan horse* effect was thus hypothesized while a tissue specific response as well as chemical interaction with n-TiO_2_ and specific chemicals could be inferred. Similarly, enhanced uptake and toxicity of an organic pollutant such as PhE in the ark clam *Scapharca subcrenata* was observed in the presence of n-TiO_2_ [114]. On the contrary, co-exposure with n-TiO_2_ did not alter the bioaccumulation of several Polybrominated diphenyl ethers (PBDE) congeners in the same species [114]. Furthermore, in a recent study conducted on the brine shrimp *Artemia salina* and n-TiO_2_ combined with PhE and Cd^2+^, the authors found that the toxicity of the pollutants was significantly affected by the presence of n-TiO_2_ [110]. On the contrary, in another study with *Mytilus edulis* the presence of n-TiO_2_ caused a significant reduction in benzo(a)pyrene (B(a)P) bioavailability, validated by lower levels in exposure water and whole soft tissue of the mussels upon co-incubation with n-TiO_2_ [115]. The presence of n-TiO_2_ has also been shown to significantly increase the embryotoxicity of tributyltin in the abalone *Haliotis diversicolor* [116].

Concerning the few contributions made on testing interactions with CECs, co-exposure of the ark clam *Tegillarca granosa* to 17β-estradiol and n-TiO_2_ significantly altered the immune system and led to a significant increase in the content of alkali-labile phosphate in the hemolymph, suggesting an enhanced 17β-estradiol bioconcentration in the presence of n-TiO_2_ [117]. As far as marine fish species, effects on liver bioconcentration/bioaccumulation of TCDD were observed in juveniles of the European sea bass (*Dicentrarchus labrax*) in vivo exposed (7 d) to the mixture (i.e., TCDD and n-TiO_2_) compared to the single chemicals, although higher levels of total Ti were detected in the presence of TCDD [118]. We hypothesized that, once in seawater, the TCDD partitioned directly into the fish without being affected by the presence of n-TiO_2_. Transcriptomic profile on phase 0, I and II of biotransformation in the liver suggested that n-TiO_2_ is unlikely to interfere with TCDD-dependent biotransformation gene expression, although the effects of the co-exposure observed in mRNAs of ATP-binding cassette transporters might suggest an impact of n-TiO_2_ on xenobiotic metabolite disposition and transport in the liver of *D. labrax* [119].

The experience gathered in nano-ecotoxicology through the last 20 years underlines the importance to define standardized protocols which include a detailed physical chemical characterization of ENMs in seawater to move towards more realistic exposure assessment studies. To this aim, the data acquired until now have been used to:

- Understand the interplay between ENMs, water media components and exposed organisms;
- Investigate *Trojan horse* mechanisms with existing marine pollutants [107,110];
- Build ENM fate and transport models and identify major receiving compartments to which concentrate major efforts in terms of protection and eventual remediation actions [9].
- Set exposures in mesocosms setting [120,121].

## 3. Current Knowledge on ENMs Marine Ecotoxicity

The use of NP suspensions has transformed the concept of toxicity testing of chemicals and recently overcame standardized methods [122,123,124] with new shared procedures for ENMs screening.

The remarkable biological, chemical, and physical reactivity of ENMs determines their environmental transformations and allows them to interact with marine organisms through different exposure routes, including direct/indirect ingestion [21,125], contact with gills [126,127] and/or body adsorption [34,128,129].

**Figure 5:**
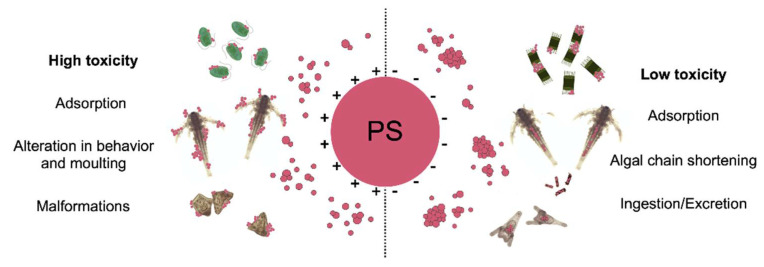
Behavior and major known toxic effects of PS NPs having positive (**left**) and negative (**right**) surface charge to model marine plankton (microalgae, brine shrimps and sea urchin larvae). Not to scale.

In marine phytoplankton, the physical adsorption of ENM agglomerates to cell membranes has been associated with several negative outcomes beyond standardized end-points such as inhibition of algal growth. For example, in unicellular microalgae and colonial diatoms, NP adsorption was related to alteration in photosynthetic activity and algal buoyancy as well as ROS production [130,131,132] (Figure 5). In marine zooplankton, most studies correspond to acute toxicity tests in short-term exposure scenarios, conducted at high concentrations of ENMs (10–100 µg mL^−1^) well above predicted environmental concentrations (PECs) for marine waters (ranging from ng L^−1^ for AgNPs to µg L^−1^ for n-TiO_2_ and nanoplastics) (reviewed in [31]). Therefore, the obtained EC/LC_50_ values for regulatory purposes were not sufficiently representative of real exposure scenarios for the ENMs investigated. For this reason, there has been an increasing interest to include prolonged exposure times at lower concentrations and investigate sub-lethal effects to discern specific modes of action that could have been hidden by standardized acute tests.

**Figure 6:**
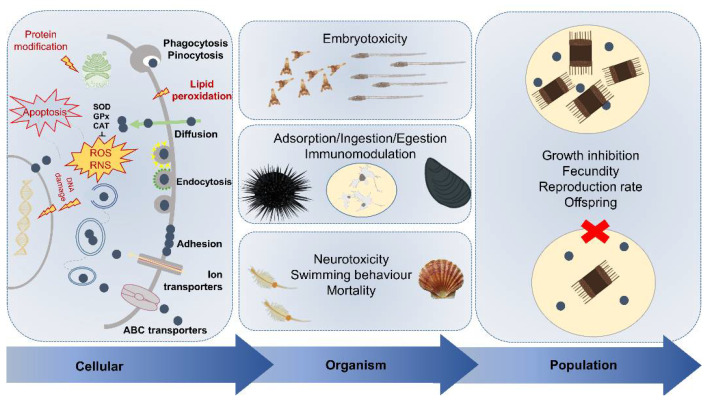
Conceptual representation of the known toxicological responses to ENMs across marine model species and at different levels of organization, ranging from single cells (**left**), individuals (**middle**) up to populations (**right**). ENMs are displayed as dark blue dots.

However, the selection of which effect to evaluate can be challenging since it varies among species, dose, duration of the exposure, type of ENMs and how these exert toxicity on the biological target [133,134] (Figure 6). Here, we report representative case studies on marine invertebrates performed in the last 10 years, showing how exposure to major types of ENMs reaching the marine environment (e.g., n-TiO_2_, PS NPs as proxy for nanoplastics and AgNPs) has been related to a range of negative effects, from cell immunotoxicity to individual neurotoxicity, alterations in development, growth and survival up to early warnings for ecosystem-scale impacts.

### 3.1. Cellular Uptake and Immunotoxicity

At the cellular level, uptake of ENMs is characterized first by cell adhesion, followed by internalization, as widely described in human cell models [134]. The physical chemical properties of ENMs and the surrounding eco-/bio-coronas govern the recognition by membrane receptors, thus regulating their uptake and toxicity [135]. ENMs can be internalized through different pathways, which are mostly energy-dependent process: (i) phagocytosis, (ii) endocytosis (clathrin-mediated, caveolae-mediated, clathrin-caveolae independent), and (iii) micropinocytosis [136,137,138]. Alternatively, they may also diffuse through the lipid bilayer [139,140] (Figure 6).

In marine organisms, endocytic pathways are generally the most employed routes for ENMs to enter cells, as reported in mussel immune and hepatopancreas cells and also for crossing the gut epithelium [141,142,143,144,145]. Once inside the cells, NPs have been found to increase ROS levels through direct catalytic mechanisms, producing highly reactive hydroxyl (•OH) radicals, or indirect mechanisms, controlling intracellular ROS generation pathways such as the mitochondrial electron transfer chain [146]. The overproduction of ROS can involve oxidative damage and influence different cellular mechanisms resulting in cytotoxicity and genotoxicity up to apoptosis and cell death [147]. In addition, the main genotoxic effects may be related to a direct interaction of ENMs with the nuclear membrane and DNA inducing various DNA damages [148].

Cell-mediated immune response has been identified as the first target of ENMs-exerted toxicity in aquatic organisms [149]. Among marine invertebrates, the sea urchin *P. lividus* and bivalves belonging to genus *Mytilus* spp., have been proposed as suitable models to study NP-cellular interactions and immunotoxicity [136,150,151], as previously adopted to study other legacy marine pollutants as well as other stressors, including seawater acidification, UV-radiation and microplastics [152,153,154,155]. Several harmful effects of PS NPs have been reported in mussel hemocytes both in vitro and in vivo, and all of these highlighted the nano-immunomodulation features [156,157,158]. Studies performed with short-term primary cultures of coelomocytes exposed to functionalized PS NPs showed that the immune response exerted by sea urchin coelomocytes differed according to PS NP surface charge. In particular, PS-NH_2_ (50 nm; 5, 10, 25 µg mL^−1^) provoked apoptotic-like nuclear alterations, reduction in cell viability and caused destabilization of the lysosomal membranes of coelomocytes [104]. Conversely, PS-COOH (60 nm; 5 and 25 µg mL^−1^) showed a less acute toxicity towards immune cells even if at the highest concentration affected phagocytic capacity in similar due to the fast internalization of PS-COOH by sea urchin phagocytes [159]. Similar effects on immunological parameters have also been reported in the Antarctic sea urchin *S. neumayeri* coelomocytes after short-term in vitro exposure to both PS-COOH (60 nm) and PS-NH_2_ (50 nm; 1 and 5 µg mL^−1^) [45] corroborating how sea urchin coelomocytes can be a formidable tool for assessing the immunotoxic effects of ENMs in marine invertebrates (Figure 6).

### 3.2. Neurotoxicity

Increasing concerns have been raised over potential neurotoxic effects of ENMs, which can interfere with the neural signal transduction of neurotransmitters. Although the mode of action remains almost unclear, ENMs have been shown to enter the central nervous system, alter the activity of Cholinesterases (ChE) and impair Acetylcholinesterase (AChE) by exerting a range of neurotoxic effects. In the marine scallop *Chlamys farreri*, an increase in AChE activity in gills and digestive gland was observed after 14 days of exposure to n-TiO_2_ (1 mg L^−1^) [160]. In contrast, Guan and co-authors [161] reported an increase in the concentrations of neurotransmitters (dopamine, γ-aminobutyric acid and ACh) and inhibition of AChE activity in the marine bivalve *Tegillarca granosa* upon in vivo acute exposure to n-TiO_2_ (0.1, 1, 10 mg L^−1^). Similar observations were reported in the Mediterranean clam *Ruditapes decussatus* exposed to n-TiO_2_ and n-Au-TiO_2_ (50 and 100 μg L^−1^) [162] and in the brine shrimp *A. franciscana* after short- and long-term exposures to PS-NH_2_ (50 nm; 0.1, 1, 3 and 10 µg mL^−1^) [35]. Furthermore, in embryotoxicity studies developmental malformations caused by ENMs have been related to changes in ChEs, since these enzymes have a role in the neuronal differentiation during development [163]. For example, in *P. lividus* embryos, the activity of the AChE results was decreased by n-TiO_2_ (10–30 nm; from 0.0001 to 1 mg L^−1^), suggesting that these NPs may cause neurotoxic damage [164].

### 3.3. Behavioral and Developmental Effects

Several behavioral and developmental effects have been reported in marine invertebrates upon exposure to ENMs. In the marine copepod *Paracyclopina nana*, PS NPs (50 nm; 10 µg mL^−1^) have been shown to affect its feeding behavior, being retained in the digestive gland. In addition, the fecundity and the growth rate were negatively affected, suggesting repercussions at population level [165]. On the contrary, cationic PS NPs have been reported to affect growth rate, fecundity, lifespan and reproduction of the rotifer *Brachionus koreanus*, with the activation of clear stress response molecular pathways [166]. Similarly, we observed a significant gut retention of PS NPs (PS-COOH 40 nm; PS-NH_2_ 50 nm; from 0.5 to 50 µg mL^−1^) in *B. plicatilis* regardless of surface charges (0.5–5 µg mL^−1^) but only cationic PS-NH_2_ NPs caused lethality to rotifer larvae and at low concentrations in reconstituted artificial seawater compared to the natural medium (LC_50_ = 2.75 ± 0.67 and 6.62 ± 0.87 μg mL^−1^, respectively) [23].

As far as developmental effects are concerned, embryotoxicity test using free spawning organisms has largely been used to assess the effects of ENMs on embryonic/larval stages of marine invertebrates, including benthic ones. Investigations on the percentage of normally developed larvae at increasing ENM concentrations and species-specific time of exposure as well as description of embryo/larvae abnormalities have been performed. Developmental stages are known to be the most sensitive to environmental and anthropogenic perturbations including to CECs, thus influencing species fitness and survival [167].

Impairment in shell formation and delay in the development have been reported in *M. galloprovincialis* embryos exposed to 50 nm PS-NH_2_ (from 0.001 to 20 µg mL^−1^) [168] and 21 nm AgNPs (100 µg L^−1^) [169]. Molecular changes in biomineralization, immune response and multixenobiotic resistance mechanism have been documented upon exposure to PS-NH_2_ NPs [168]. Similar sub-lethal effects have also been reported in embryos of the oyster *Crassostrea gigas* exposed to PS-NH_2_ (50 nm; from 0.1 to 25 µg mL^−1^) leading to major developmental effects over plain PS NPs (50 nm, 500 nm and 2 µm; from 0.1 to 25 µg mL^−1^) and PS-COOH (50 nm; from 0.1 to 25 µg mL^−1^) such as mantle, shell and hinge malformations up to full developmental arrest [32]. More recently, we reported the disruption in the development of the ascidian *Ciona robusta* embryos associated with oxidative stress and various degree of malformations in larvae phenotypes upon exposure to PS-NH_2_ (50 nm; from 2 to 15 µg mL^−1^) between 10 and 50 µg mL^−1^ [128]. In the sea urchin *P. lividus* embryos, dose-dependent morphological alterations, including skeletal anomalies, body asymmetry or arrested development, have been reported upon exposure to several ENMs [164,170,171,172] such as AgNPs (average diameter: 5–35 nm; 0.03–3 µg mL^−1^) [173] and PS NPs [22,174] in the range 0.1–10 µg mL^−1^. At the molecular and biochemical level, first insights into the signaling pathways related to 50 nm PS-NH_2_ exposure showed the modulation of proteins and genes involved in stress response and development (Hsp70, p38 Mapk, Caspase 8, Univin) at the highest concentrations (<10 µg L^−1^) [174] (Figure 6).

### 3.4. From Impact on Single Species Up to Populations and Communities

The exposure to ENMs and in particular to polymeric NPs as proxy for nanoplastics has often revealed sub-lethal effects which are usually overlooked as end-points in standardized ecotoxicological tests, although they could provide important insights on possible ecological implications in natural exposure scenarios. Even if the core constituents and the surface chemistry play an important role in establishing the acute toxicity of polymeric NPs, their physical impact, as surface adhesion or gastric hindrance following their ingestion, could lead to severe effects under chronic exposure (Figure 5).

In our recent study on the marine diatom *Skeletonema marinoi* [129], while no toxicity upon exposure to PS-COOH NPs (1, 10, 50 µg mL^−1^) was found, a strong adhesion of NP aggregates onto cell surface associated with algal chain shortening was recorded. *S. marinoi* is organized in chains, whose purpose is hypothesized to be linked to an improved buoyant capacity, which allows the algal bloom to persist longer in the euphotic zone. A reduction in chain length might result in a faster sinking of the algae and a reduced growth of the bloom. The interaction of microorganisms and their exudates with plastics was observed to affect the sinking rates of phytoplankton aggregates, with related potential consequences on biogeochemical cycles and carbon fluxes [175]. Furthermore, our study highlighted the interaction of PS NPs both with the algal surface and with algal exudates, which, when considered in an ecological perspective, may pose a hazard both in terms of trophic transfer and of biofilm formation. In laboratory settings, an effect such as chain length reduction is easily overlooked as no direct negative effect is evident. However, this apparently negligible effect could mean a possible disruption of an ecological function and of the many life forms that depend on it. Another example comes from our study [34] in which we observed the adhesion of positively charged PS-NH_2_ (5–100 µg mL^−1^) to the body surface of microcrustaceans associated with an increase in the molting events in the 48 h, but no mortality. However, within a longer exposure period (14 d), a pronounced reduction in growth and mortality of the brine shrimp *A. franciscana* were observed, and probably linked to the higher energy demand caused by multiple molting in the attempt of getting rid of the particles from the surface of the body causing a mechanical disturbance [21]. This highlights also the importance of focusing on long-term testing to obtain more environmentally relevant data and avoid the risk of underestimating the potential threat posed by ENMs to marine ecosystems. In our last work on AgNPs ecotoxicity upon short and long-term exposure scenarios [176], this concept came out clearly: the acute exposure (48 h) of *A. franciscana* to AgNPs resulted in the total lack of toxic effects up to extremely high concentrations (100 µg mL^−1^), although ingestion of AgNPs was observed. Upon chronic exposure (14 d), a 100% mortality was then recorded already at 10 µg mL^−1^ AgNPs, with a shift in the EC_50_ from >100 µg mL^−1^ (48 h) to 5.087 µg mL^−1^ (14 d). In future nano-ecotoxicity studies, more sub-lethal effects which are linked to impacts on population should be included as well as prolonged exposure scenarios in order to mimic real exposure conditions.

Effect concentrations can be put into perspective by considering PECs and field measurements currently available for selected ENMs. AgNPs and n-TiO_2_ ending up in the aquatic environment, mainly from wastewater treatment plants (WWTPs), have been estimated to reach influents at concentrations in the range from µg L^−1^ to ng L^−1^ [177,178,179]. A study by Nabi and co-authors [178] on five WWTPs in the United States, measured a high removal efficiency towards AgNPs (82–95%) and n-TiO_2_ (90–96%), with an effluent concentration range of, respectively, 0.008–0.04 µg L^−1^ and 6.9–30 µg L^−1^. Previously, Li et al. [179] also reported a high removal efficiency for AgNPs in a WWTP in Germany (up to 96.4%), but the estimated annual release in the effluent was still high (~33 Kg of AgNPs). The smaller particles appear to be able to escape the treatment process, being consequently released in the effluent, with an overall reduction in NP size from influent to effluent. Cervantes-Avilès et al. [177] report a reduced average size of AgNPs, from 100–200 nm to 50 nm, while Nabi et al. [178] report that more than 99% of AgNPs and from 55 to 97% of n-TiO_2_ in effluents are smaller than 100 nm. A reduced size has often been associated with a higher risk of cellular internalization and whole organism toxicity [180,181]. For instance, in cellular exposure, the degree of agglomeration and thus the overall size of AgNPs was correlated to a different cellular localization, with smaller particles-aggregates being able to reach nuclear and mitochondrial compartments [180]. Concerning n-TiO_2_, effect concentrations (EC_50_ range µg L^−1^–mg L^−1^) might fall within the range of the reported wide PECs for the marine environment since local sources associated with specific uses (e.g., sunscreens, antifouling paints, oil spill pollution remediation technologies) could significantly increase the mass input into surface waters of marine coastal areas and deep sediments [9]. For instance, levels of Ti in marine coastal bathing waters, attributed to their presence as UV filters in sunscreen products, fall into the range of 100–900 μg L^−1^ in the top surface layer down to 20–50 μg L^−1^ in the water column and <5 μg L^−1^ far from the bathing zone [182]. However, the strong aggregation process which n-TiO_2_ undergo in marine waters driven by salts and NOM (e.g., homo and heteroagregation) could significantly reduce its bioavailability and then toxicity for marine species in such natural systems as already reported in several studies [31]. Therefore, it is mandatory to link exposure conditions to the observed biological effects and thus support the need to characterize the NP behavior in the natural medium for a more realistic risk assessment. As far as AgNPs, although PECs (≤µg L^−1^) [183,184] and reported effect concentrations (EC_50_ range from 1 µg L^−1^ to >100 mg L^−1^) [83,87,181,185,186,187,188,189,190] rarely overlap, the increasing AgNP use and production such as, for instance, as sanitizing agents and antimicrobial textiles, also linked to the current sanitary emergency of COVID-19 worldwide pandemic [191,192], could easily contribute in the near future to an increase in the PECs and associated risks [193]. Considerations should be also made for the extremely wide effect concentration range for AgNPs (1 µg L^−1^–>100 mg L^−1^); this is due to their many different features and to the high variability of exposure conditions, both deeply influencing their behavior and toxicity. However, it is also caused by the large discrepancy between short-term toxicity exposure and environmentally relevant chronic exposure scenarios [84,194], posing uncertainties for a realistic AgNPs risk assessment [195]. Finally, regarding nanoplastics, reported effect concentrations for marine species appear to vary according to PS NP functionalization, with high E(L)C_50_ (usually >100 mg L^−1^) reported for plain and negatively charged NPs and low E(L)C_50_ values (range 0.10–10 mg L^−1^) for positively charged NPs upon acute exposure (reviewed in [31,196]). Through a species sensitivity distribution analysis of PS NPs effects on marine organisms, the hazard concentration for 5% of the species (HC_5_, that is a safe value for 95% of the species considered) has been estimated equal to 0.41 mg L^−1^ [197]. These effect/hazard concentration values are far above PECs of nanoplastics in natural environments, estimated between approx. 1 pg L^−1^ and 20 μg L^−1^ based on Lenz et al. [198]. However, environmental data regarding nanoplastics occurrence and distribution in marine compartments are still extremely limited and real concentrations may exceed conservative PECs in regions characterized by high levels of plastic pollution, as coastal marine areas in the Mediterranean Sea [199], approaching toxicity thresholds.

## 4. Gaps to Be Filled

### 4.1. Real Exposure Scenarios: Nano-Enabled Products (NEPs)

To date, most nano-ecotoxicity studies have been performed using bare ENMs but it is known that they are actually released into the environment across their lifecycle (from production to usage and disposal) as NEPs [10,200,201]. The high potential for environmental exposure results particularly predominant for those NEPs in which ENMs are suspended in a liquid solution [202] since they can diffuse more easily in the aquatic environment and reach biological targets by causing toxicity [10,203,204].

In the last ten years (2010–2020), only 17 scientific papers have tested NEP ecotoxicity, while more than 6300 articles concerning NPs and ENMs have been published (Figure 3b). According to the Nanotechnology Consumer Products Inventory, metal-based NPs account for the largest fraction of NPs applied in consumer products (37%), with AgNPs as the most used (24%) [79]. The increasing production of Ag-based NEPs is related to the high variety of applications of AgNPs, which vary from sport clothing to cosmetics and medical devices [205]. Among categories of products, the textile-one is recognized as the main contributor of AgNPs released by NEP in water bodies [202,206]. However, almost no information is currently available on their commercial formulation thus making rather difficult to assess any potential risks associated with their use, disposal and release into the aquatic environment [202,207]. Therefore, more studies should be carried out to first assess the release of NPs from NEPs in environmental media and establish the physical chemical properties which will dictate their behavior and fate in the aquatic environments. This will help to predict potential risk to aquatic biota and drive future studies for a proper environmental and human risk assessment.

Our recent study performed with AgNPs-enabled commercial product names nanArgen™ (Nanotek S.A.) [208] showed adverse effects similar to those reported for bare AgNPs at environmentally relevant concentrations in the marine mussels *M. galloprovincialis* [148,209,210,211,212]. Disruption of ATP binding cassette proteins functionality in gills, damage on lysosomal membranes in hemocytes and increase in oxidative stress in digestive glands were reported. More than 80% of the commercial products was confirmed to be made of Ag and the presence of well-defined and well-dispersed roughly spherical AgNPs was confirmed in MilliQ while a strong agglomeration was observed in seawater as reported for bare AgNPs [148]. Furthermore, a significant concentration-dependent accumulation of Ag was also found in mussels’ whole soft tissue in agreement with previous observations [148] in the same species upon exposure to AgNPs. Therefore, NEPs should be a focus of future nano-ecotoxicity studies being more relevant for assessing real exposure scenarios and predicting ENMs-induced risks to marine biota. Biological targets of NEPs can be diverse according to product typology, use and disposal into the marine environment and in particular those ending up in sewage effluents indeed could cause serious hazards to marine coastal areas. Last but not least, worst exposure scenarios could also cause accumulation of Ag in shellfish and fish products with possible transfer to humans through consumption of seafood.

### 4.2. Impact on Benthic Species Overlooked

Marine benthos play a central role in marine ecosystems functioning and services. Benthic communities largely differ based on type of substratum, depth and dynamics of carbon and water flow [213], all parameters affecting behavior, bioavailability and toxicity of ENMs once reaching the sea floor [214].

Over the last 10 years, the studies on the impact of ENMs on marine benthos mostly focused on filter-feeders such as marine mussels, whereas other species representative of marine benthic communities have been poorly represented. Here, we report the contributions available on ENMs toxicity on marine benthic species other than *Mytilus* spp., from benthic prokaryotes to burrowing polychaetes, which were mainly focused on metal-based ENMs such as n-TiO_2_, PS NPs and AgNPs, including the role of capping agents in driving AgNP ecotoxicity.

Antizar-Ladislao and co-authors [215] reported significant changes in microbial communities’ structure in estuarine sediments with key microorganisms such as *Pelobacter propionicus* disappearing following exposure to AgNPs. Chan and co-authors [216] described a delay in growth and development, and a reduction in larval settlement of the barnacle *Balanus amphitrite*, the slipper-limpet *Crepidula onyx*, and the polychaete *Hydroides elegans* upon chronic exposure to sub-lethal concentration of oleic acid coated AgNPs and polyvinylpyrrolidone AgNPs (AgNPs-PVP) regardless different coatings. Wang and co-authors [217] reported no toxicity to citrate (AgNPs-citrate) and AgNPs-PVP on the amphipod *Ampelisca abdita* and the mysid crustacean *Americamysis bahia* up to concentrations of 75 mg kg^−1^ d.w. However, higher Ag levels were found in the marine polychaete *Nereis virens* upon exposure to AgNPs-citrate rather than AgNPs-PVP, demonstrating the influence of surface capping agents on Ag uptake, biotransformation, and excretion. DNA damage as well as changes in phenoloxidase and lysozyme activities have been reported in two endobenthic species, the bivalve *Scrobicularia plana* and the polychaete worm *Nereis (Hediste) diversicolor* upon exposure to AgNPs [218]. Similarly, cytotoxicity and genotoxicity have been found in *N. diversicolor* exposed to AgNPs regardless of their size (20 and 80 nm) [219]. The particles were also found in the lumen of the marine ragworm, indicating a direct internalization into the gut epithelium. AgNPs were in fact associated with the apical plasma membrane in endocytic pits and in endosomes, thus an endocytic pathway appears to be a key route of cellular uptake [220,221]. Cozzari and co-authors [221] further investigated time- and concentration-dependent accumulation of Ag in *N. diversicolor* exposed to dissolved Ag, AgNPs, and bulk Ag, showing a different biomarkers response profile related to the Ag form.

In our previous study on the benthic foraminifera *Ammonia parkinsoniana* exposed to different ENMs, we found evidence of n-TiO_2_ and PS-COOH (1 µg mL^−1^) internalized upon short-term exposure (48 h) [222]. The presence of the ENMs in the cytoplasm of this bottom-dwelling organism was associated with an increase in neutral lipids and ROS production. Moreover, n-TiO_2_ has been reported to cause immunotoxicity and neurotoxicity in the bivalve *Tigellaria granosa* [117,161] and transferred along marine benthic food chain. Wang and co-authors [223] also described an accumulation of n-TiO_2_ in juvenile turbots (*Scophthalmus maximus*) fed with n-TiO_2_-exposed clam worm *Perineris aiubithensis*. Although no biomagnification was documented, the accumulated n-TiO_2_ in turbot caused a reduction in nutrition quality and growth, and liver and spleen damage.

Regarding PS NPs, only few studies documented their impact on marine benthos upon short-term exposure and at high concentrations, far above those reflecting relevant exposure scenarios for marine waters (i.e., PEC approx. 1–10 µg L^−1^). PS NPs aggregates have been found in *N. diversicolor* [224] associated with changes in cholinergic function (ChE activities) and in borrowing behavior with potential consequences on ecosystem functioning due to their role in promoting substrate oxygenation as well as resuspension and distribution of nutrients and contaminants [225].

Such a limited number of studies underlines the current difficulties in investigating the impact of ENMs on benthic species living in strict contact with marine sediments [226]. In addition to marine mussels and sea urchins, which have widely been adopted to study ENMs ecotoxicity (see Section 2), polychaeta are by far the most used alternative model organisms for marine benthos. This is probably due to their suitability to be kept and tested under controlled laboratory conditions, which is linked to their recommended use in standardized bioassays by environmental agencies (e.g., OECD). Overall, the current state of the art fails to represent the diversity and complexity of the marine benthic species which may be exposed to ENMs in real marine environments. Our recommendation for future studies is to overtake the use of standard model organisms in order to increase the knowledge on the impact of ENMs on benthic communities.

## 5. A Lesson to Learn: Using Ecotoxicity for a Safer ENMs Design (Eco-Design)

**Figure 7:**
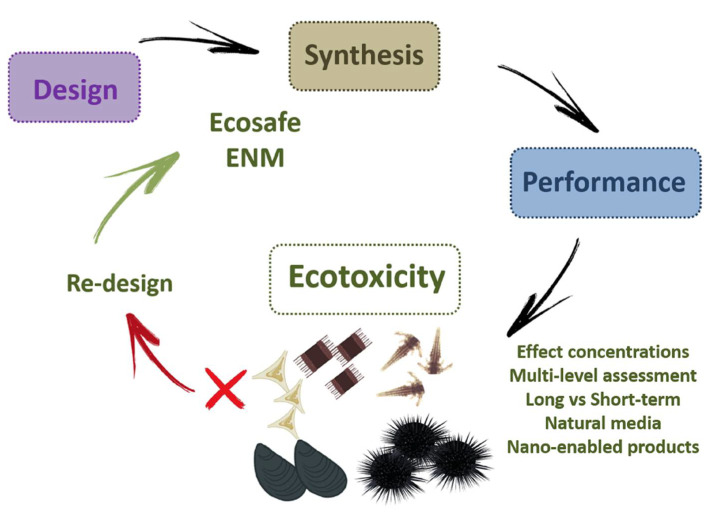
The proposed eco-design framework of ENMs. Ecotoxicity should be included in the design framework together with performance from synthesis to production. Reprinted from ref. [231].

According to the principle of “Green Nanotechnology”, progress towards safer ENMs can be made including the safety aspects in the design of ENMs (e.g., safe by design) and testing potential hazards posed by their release and application following the fundamental and well- developed framework of legacy pollutants [227]. In this view, only those properties of ENMs which will guarantee human and environmental safety while maintaining their efficacy towards various applications should be incorporated in the material design (and re-design) process during the product development. This means that ecotoxicity should be tested since the beginning of the ENM design and included among the validation framework together with its efficacy in the product development before being put into the market. The incorporation an ecotoxicity testing strategy will allow not only to re-design already existing ENMs which have been shown detrimental effects on living organisms but to define the way they should be manipulated from the beginning up to the end of their life cycle [228,229]. The development of new ENMs, for instance, those able to remove organic and inorganic contaminants and reduce their bioavailability in water media (e.g., nanoremediation), currently does not imply a testing framework for their environmental safety (eco-safety) in terms of effects for marine biota [230]. By including ecotoxicity in the testing framework, effect concentrations can be obtained and merged with efficacy concentrations of the ENM itself in order to reduce/limit any risk associated with their field application. Whether analytical chemistry is necessary to detect the concentration of contaminants in seawater and to determine the remediation efficiency of the developed ENM, it is not enough to well characterize its potential side effects in terms of environmental impact. Instead, ecotoxicological tools aiming to assess the risk for marine species could fulfil such needs and should be incorporated into ENM design framework (eco-design) [231] (Figure 7).

Although such a “safety precautionary strategy” has not yet been put in place and only recently promoted in EC funded projects together with human safety (www.nanosafetycluster.eu, accessed on 17 June 2021), few contributions already show promising results.

A sorbent cellulose-based nanosponge (CNS) material was developed by Melone and co-workers [232], using TEMPO-oxidized cellulose nanofibers (TOCNFs) and branched polyethyleneimine (bPEI) as the cross-linking agent. The high adsorption capability of this material was verified for different organic pollutants [233] and heavy metal ions [229], indicating their potential for water decontamination, owing to a highly porous structure. Moreover, these characteristics combined the utility of high surface area to volume ratio typical of ENMs with the advantages of a macroscopic material, hence avoiding the direct release of nanosized objects in natural waters. In order to verify the eco-safety of such sorbent materials, the original batch underwent ecotoxicity testing and the results fed back the synthetic process and the tuning, in order to reach an eco-safe final product. Standardized ecotoxicity assays, such as algal growth inhibition tests (OECD 201) with the marine microalga *D. tertiolecta* [229] revealed a substantial decrease in cell viability under different simulated usage scenarios, mimicking envisaged in situ situations, proving the material unsuitable for an environmental use. A potential release of bPEI from the tested material in water was identified as causing the observed effects. bPEI is a polycationic molecule that can cause cell toxicity mainly through electrostatically favoured interactions with biological membranes [234,235]. In order to overcome the issue of bPEI release and its toxicological side-effects, the synthesis of the CNS material was tuned, and the purification protocol was optimized following a step-by-step eco-design scheme combining ecotoxicological assays and chemical analysis. In brief, citric acid was added to the formulation in order to improve the immobilization of bPEI in the nano-structured network of sponges, maintaining the eco-sustainability characteristics of the material itself, and the post-synthesis washing procedure ameliorated to efficiently remove unreacted chemicals. Different batches of CNS were again characterized using algal growth inhibition tests (OECD 201) but the trophic level was amplified by pairing such assays with in vivo exposure of the materials using marine bivalves [229]. In this way, we selected the best material batch option, able to remove several toxic metal ions from seawater and combining the evidence of non-toxic effects toward phytoplankton and filter-feeders [229]. Consequently, in situ nanoremediation process was mimicked in the laboratory using ZnCl_2_ as a model marine pollutant [236]. Specimens of *M. galloprovincialis* were in vivo exposed to Zn-contaminated seawater previously treated with CNS, but also to CNS alone, to verify any potential side effect of the material itself. The ability of CNS to carry out their remediation action in terms of reducing the bioavailability of Zn to mussels was demonstrated by the limited Zn induced-chromosomal damage and lysosomal membrane destabilization in mussels’ hemocytes. The eco-safety and the sorbent properties of the material were tested also at the tissue level. Morphological alterations resulted particularly evident in the mantle of organisms exposed to Zn-contaminated waters, while an intact (and equal to controls) mantle edge morphology occurred in specimens exposed to the contaminated water treated with CNS and CNS alone. Additionally, we noticed a continuous and unrestricted release of mucus secretion, which is considered a typical stress response in mussels, only in specimens exposed to Zn, while in pristine and CNS-treated seawater mucus cells were not secreted. Moreover, Zn(II) levels in mussel’s exposure waters were reduced to more than 90% after CNS treatment, as a further confirmation of the CNS absorption ability. Marine algal assays provided a rapid and effective screening test for the toxicity of reagents employed in the novel formulations by representing an ecotoxicological high level endpoint. The testing on marine mussels allowed to demonstrate through clear signals at cellular and tissue levels both the eco-safety and the effectiveness of the remediation of this newly nanostructured cellulosic material.

The execution of in vitro and in vivo ecotoxicological experiments with marine algae and mussels proved CNS to be effective in quickly and accurately removing toxic chemicals from waters by mimicking a real environmental scenario. Moreover, the stepwise employed procedure allowed us to follow both the pre-usage material conception and to gain information on the potential side effects of the envisaged environmental use. Another successful example comes from AgNPs, in which an eco-safety assessment promotes their safe application in mercury (Hg) sensing and remediation from seawater. The fields of application of AgNPs are growing daily and thanks to their unique properties, AgNPs can be used for many different purposes [193]. For those applications not requiring AgNPs to express their biocidal properties, for instance, environmental applications, the release of Ag^+^ ions, the major drive of toxicity of AgNPs, could be prevented by means of an ad hoc design in order to reduce their impact. The environmental costs of the end of life and disposal of ENMs should be considered when they are included in consumer products, and their potential impact on ecosystems reduced as much as possible [231].

The affinity of metal ions for molecules with reduced sulfur groups is well documented [237]. Evidence has shown that the sulfidation of AgNPs, with the formation of an insoluble Ag sulfide (Ag_2_S) layer onto the NPs surface, is able to strongly inhibit their dissolution, as well as enhancing aggregation and reducing their mobility [238]. These transformations, as well as the reduction in the amount of Ag^+^ ions that are freed in aqueous ecosystems, may also limit the environmental impact of AgNPs by promoting their sedimentation and reducing their reactivity. The sulfidation of AgNPs is a process that can spontaneously take place during wastewater treatments or in natural aqueous systems, in the presence of organic and inorganic sulfur containing molecules [91]. However, the sulfidation of AgNPs, or similar surface modifications, can also be obtained through the addition of a capping agent during synthesis. During the design process, surface coatings of AgNPs are usually selected based on the need to fulfil certain requisites, for instance preventing aggregation [239]. Based on the desired application, when possible, AgNPs should be designed in order to reduce as much as possible also their potential toxicity.

A few studies have investigated the toxicity of AgNPs functionalized with sulphur groups and reported how it was always lower than that of pristine AgNPs [89,90,240]. In a recent paper by Prosposito and co-workers [90], we showed how the functionalization of AgNPs with both citrate and L-cysteine was able to prevent any toxic effect for both freshwater and marine microalgae. These particles were synthesized for the remediation of Hg-contaminated waters, hence their safety for the water environment needed to be assessed as the primary issue. This type of coating (i.e., citrate and L-cysteine-based) demonstrated to be very efficient in the binding of free Hg^2+^ ions, while at the same time being able to lock Ag^+^ ions in the NPs core, with no release of toxic species and no biological effect. The release of Ag^+^ ions in solution as well as their ecotoxicity was determined in two model microalgae, *R. subcapitata* from the freshwater environment and *P. tricornutum* from the marine environment. The AgNPs functionalized with the double coating of citrate and L-cysteine proved to be totally safe both in terms of ion release and ecotoxicological effects, in the two tested environmental conditions.

## 6. Concluding Remarks and Future Perspectives

ENMs are indeed highly dynamic in seawater and a full detailed physical chemical characterization of their acquired properties is mandatory for a proper assessment of exposure scenarios and related environmental and human risks.

Eco-interactions between ENMs and (bio)molecules, including existing chemical pollutants, define new exposure scenarios that need to be taken into account when approaching the (eco)toxicity study of nanosized objects, as they interact with living entities in a fundamentally different way than classical contaminants. Therefore, the eco-corona concept needs to be incorporated into the ecotoxicological perspective of environmental nano-bio interactions. In terms of biological effects, more sublethal end-points, which will bridge the impact from single organism to population and communities, should be included as well as prolonged exposure conditions in order to mimic real exposure scenarios. A closer look to NEPs is also strategically needed, being more relevant for assessing real exposure to commercial products containing ENMs and predicting hazards for marine biota. Our recommendation for future studies is also to overtake the use of standard model organisms in order to increase the knowledge on the impact of ENMs on benthic communities. An ecologically based safer by design strategy (eco-design) is thus proposed, in which an ecotoxicological testing strategy will allow the selection of the best eco-friendly and ecologically sustainable ENMs, thus significantly limiting any potential future side effects in terms of toxicological risk for natural ecosystems including the marine environment.

## Data Availability

The data presented in this study are available within the article.
